# Supplementary material for: The M35 Metalloprotease Effector FocM35_1 Is Required for Full Virulence of Fusarium oxysporum f. sp. cubense Tropical Race 4
Source: Pathogens. 2021 May 29;10(6):670. doi: 10.3390/pathogens10060670 (PMC8226822; doi:10.3390/pathogens10060670)
Supplement: Supplementary file 1 [file pathogens-10-00670-s001.zip › supplementary files/supplementary caption.pdf]

Supplemental Table S1. Primers used in this study.

Supplemental Figure S1. Multiple sequence alignment of M35 domain and its homologues mainly from *Fusarium* spp. Secondary structures inferred by ESPrint 3.0 from the PDB 3D structure of P46076 are denoted above the coordinates.

Supplemental Figure S2. Functional test of the predicted signal peptide of FocM35_1 using a yeast secretion system. Upper image shows fusion of the functional signal peptide of FocM35_1 with invertase enables the yeast strain grown on a CMD-W agar plate (without tryptophan). Lower image shows the reactions with triphenyltetrazolium chloride (TTC) solution; a color change indicates the conversion of TTC to triphenylformazan catalyzed by secreted invertase. The known functional signal peptide Avr1b was used as a positive control, and the non-secreted peptide Mg87 was used as a negative control.

Supplemental Figure S3. Schematic representation of gene disruption strategy and Southern blotting analyses of the deletion mutant. (a) Schematic representation of the FocM35_1 disruption strategy. (b) PCR confirmation results of gene disruption and (c) complementation. (d) Southern blotting assays. A fragment of hygromycin resistance gene (HPH) was used as the probe.

Supplemental Figure S4. FocM35_1 is required for successful penetration and colonization and is involved in the response towards oxidative and osmotic stress. (a) Penetration assays with cellophane membranes. Wilt type (WT), ΔFocM35_1 and ΔFocM35_1-Cstrains were grown for 3 days on top of cellophane membranes placed on minimal medium (before). The cellophane membranes were removed, and the plates were incubated for 2 additional days to examine hyphal growth (after). (b) Comparison of the infection behavior of WT, ΔFocM35_1 strain on banana roots with scanning electron microscopy. The penetration event is indicated by red arrows. The areas within the red squares in the left panels are enlarged into pictures in the right panels. (c) Sensitivity assays against NaCl and H_2O_2. Colony morphology of the WT and ΔFocM35_1 strains are shown after 5 days of incubation at 28°C on MM plates or MM plates supplemented with 1.5 M NaCL or 20 mM H_2O_2. (d) Statistical analysis of the growth inhibition of strains under osmotic stress and oxidative stress shown in c. Statistically significant differences were determined by Student's t test (* $p < 0.05$).

Supplemental Figure S5. Purification of the recombinant FocM35_1.

Supplemental Figure S6. Clustering analysis of the expression patterns of banana chitinase genes encoding proteins with GH19 domain, based on RNA-Seq data from banana roots inoculated with *Foc* TR4. Gene expression level is present as the log2 transformation of the original fragments per kilobase of transcript per million mapped reads (FPKM) value, and the values are shown for different phase of infection. The size and the color of each filled circle indicated the gene expression level.
