# Supplementary material for: The M35 Metalloprotease Effector FocM35_1 Is Required for Full Virulence of Fusarium oxysporum f. sp. cubense Tropical Race 4
Source: Pathogens. 2021 May 29;10(6):670. doi: 10.3390/pathogens10060670 (PMC8226822; doi:10.3390/pathogens10060670)
Supplement: Supplementary file 1 [file pathogens-10-00670-s001.zip › supplementary files/Table S1 Primers used in this study.pdf]

Table S1. Primers used in this study

Name	Sequence (5’-3’)
FOIG-01919^{UP}-F	GTGGTAGTGTGGAGGGGC
FOIG-01919^{UP}-R	CAAAATAGGCATTGATGTGTTGACCTCCTTTGAACGATAGACCAGATCGAACG
FOIG-01919^{DOWN}-F	CTCGTCCGAGGGCAAAGGAATAGAGTAGCCCAGGAGAACTCCACCGA
FOIG-01919^{DOWN}-R	CAAGTGTCAACGAGGATATCCTTG
FOIG-01919-I-F	ACACCAAGACGGATCAATATCGT
FOIG-01919-I-R	AGCAGAAGCCTGTCTAGCAC
FOIG-01919-N-F	ATCTGATACACGATAATGCCCAACC
FOIG-01919-N-R	AGCGTTAACTCGCGTACTCTAG
FOIG-01919-C-F	GTACTCAAATTGGTTCTCGAGGCTTTAGCCTATGCGAACGTTCC
FOIG-01919-C-R	GCCCTTGCTCACCATCTCGAGACATCCCAGACTAACAGCGTTGG
Hyg-F	CAAAATAGGCATTGATGTGTTGACCTCC
Hyg-R	CTCGTCCGAGGGCAAAGGAATAGAGTAG
FOIG-01919-gfp-F	GAACCCAATCTTCAAACTCGAATGAAGCTGCTTGCTGGTCTT
FOIG-01919-gfp-R	TCGAGGTGAGCAAGGGCGAGGCTAACATCCCAGACTAACAGCGTTG
qTUB-F	AGCAACTCCTACTTTTGGCGATC
qTUB-R	TGAGGGCCCTGTATTGCTGG
EF1a-F	ACACGTGTTGCTTCTGCGAA
EF1a-R	TGCAGCCGTGTCTTCTGTTC
FOIG-01919-RP27-F	GAACCCAATCTTCAAACTCGAATGAAGCTGCTTGCTGGTCTT
FOIG-01919-RP27-R	TCGAGGTGAGCAAGGGCGAGGACATCCCAGACTAACAGCGTTG
FOIG-01919-1300-F	AGCTCGGTACCCGGGGATCCTATGAAGCTGCTTGCTGGTCTT
FOIG-01919-1300-R	GGTCGACTCTAGAGGATCCCCCTAACATCCCAGACTAACAGCGTTG
FOIG-01919-vennes-F	GGTGAGCTCGGTACCAAGCTTATGAAGCTGCTTGCTGGTCTT
FOIG-01919-vennes-R	GCGGCCGCACTAGTAAGCTTCTAACATCCCAGACTAACAGCGTTG
FOIG-01919-pET28-F	gacggtaccgcgggcccgggatccaccggatctagaATGAAGCTGCTTGCTGGTCTT
FOIG-01919-pET28-R	TCGAGTGCGGCCGCAAGCTTGTAACATCCCAGACTAACAGCGTTG
FOIG-01919-noSP-F	AGCTCGGTACCCGGGGATCGCGCCCCTGGTGGATAAG
FOIG-01919-noSP-R	GGTCGACTCTAGAGGATCCCCCTACTTATCCACCAGGGGCGC
FOIG-01919-SB-F	GCTCGGTA CCCGGGATGAAGCTGCTTGC
FOIG-01919-SB-R	CAGGTCGAC TCTAGACTAACATCCCAGAC
FOIG-01919-SB-T-F	gggcaacaccaacttgcacgcgatgacccatcttaaccagatc
FOIG-01919-SB-T-R	gatctggttaagatgggtcatcgcgtgcaagttggtgttgccc
Ma09-20710-1300-F	AGCTCGGTACCCGGGGATCCTATGGCACATCAGAACAGGATGG
Ma09-20710-1300-R	GGTCGACTCTAGAGGATCCCCTAGCAAGTGAGGTTGTCTCCGG
Ma09-20710-vennes-F	GGTGAGCTCGGTACCAAGCTTATGGCACATCAGAACAGGATGG
Ma09-20710-vennes-R	GCGGCCGCACTAGTAAGCTTTTAGCAAGTGAGGTTGTCTCCGG
FOIG-01919-SP-F	TCCAAGCTCGGAATTTTAATTAAGAATTCATGAAGCTGCTTGCTGGTCTT
FOIG-01919-SP-R	ATACGACTCACTATAGGGAGAACCTCGAGCTTATCCACCAGGGGCGC
