# Supplementary figures and images for: The M35 Metalloprotease Effector FocM35_1 Is Required for Full Virulence of Fusarium oxysporum f. sp. cubense Tropical Race 4
Source: Pathogens. 2021 May 29;10(6):670. doi: 10.3390/pathogens10060670 (PMC8226822; doi:10.3390/pathogens10060670)

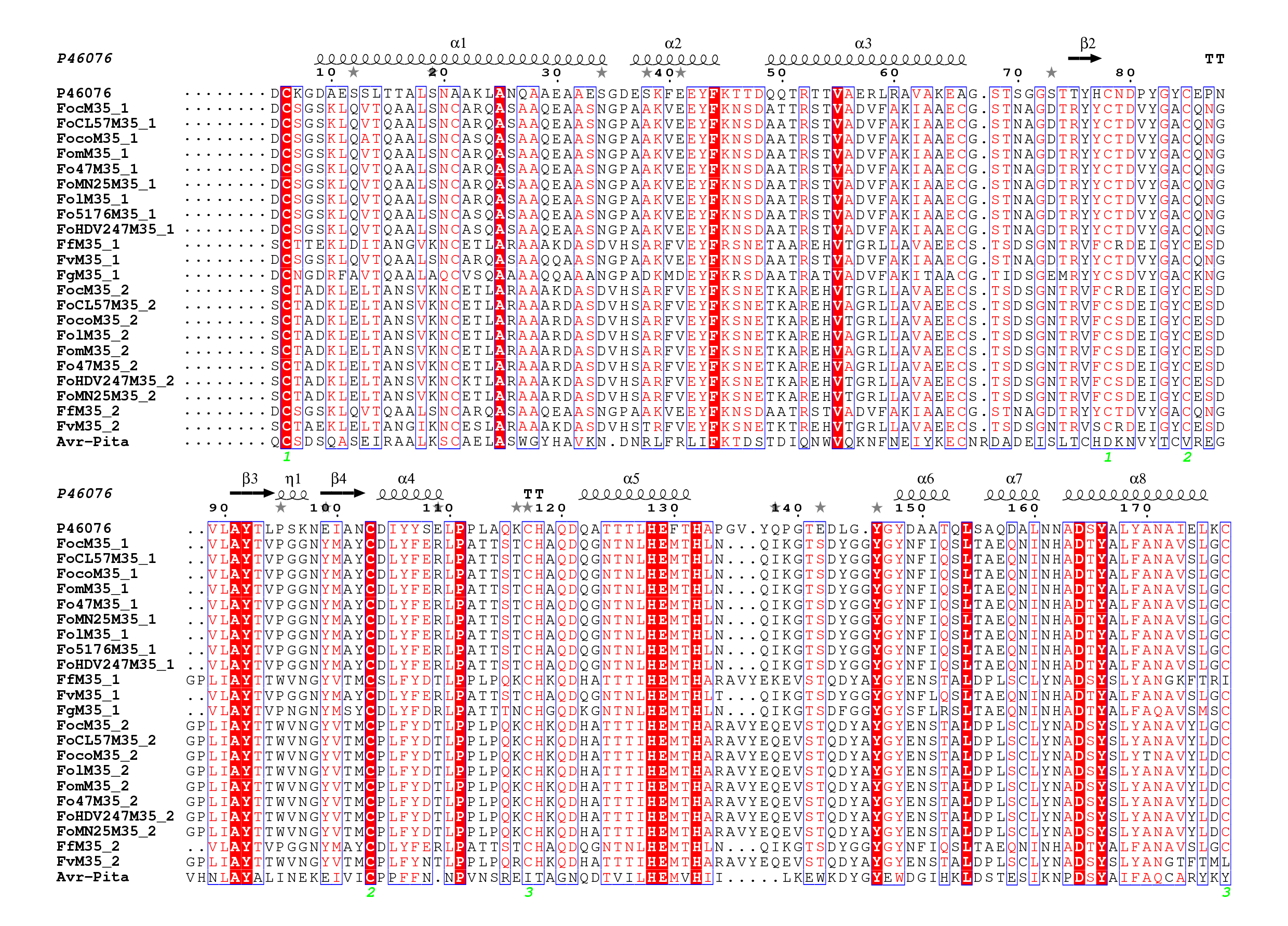

Supplement: Supplementary file 1 [file pathogens-10-00670-s001.zip › supplementary files/Figure S1. alignment and secondary structure.jpg]

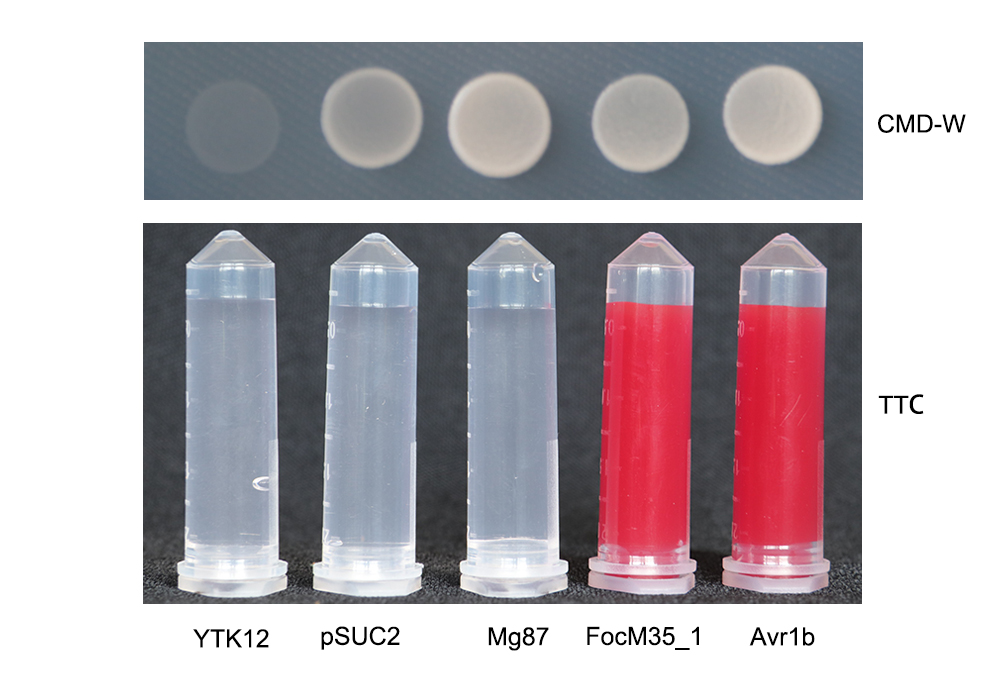

Supplement: Supplementary file 1 [file pathogens-10-00670-s001.zip › supplementary files/Figure S2. functional analysis of predicted signal peptide.jpg]

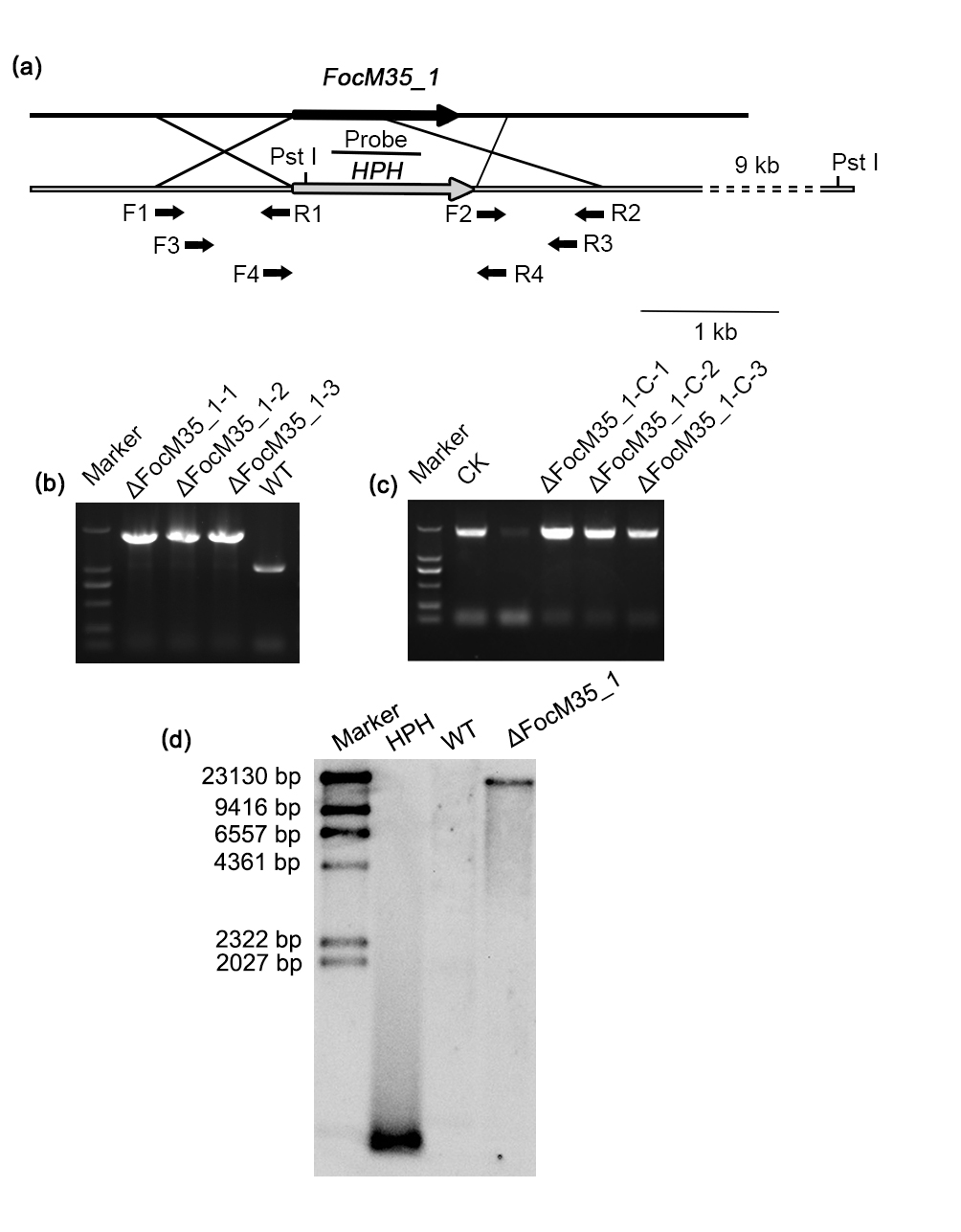

Supplement: Supplementary file 1 [file pathogens-10-00670-s001.zip › supplementary files/Figure S3. gene disruption strategy and Southern blotting.jpg]

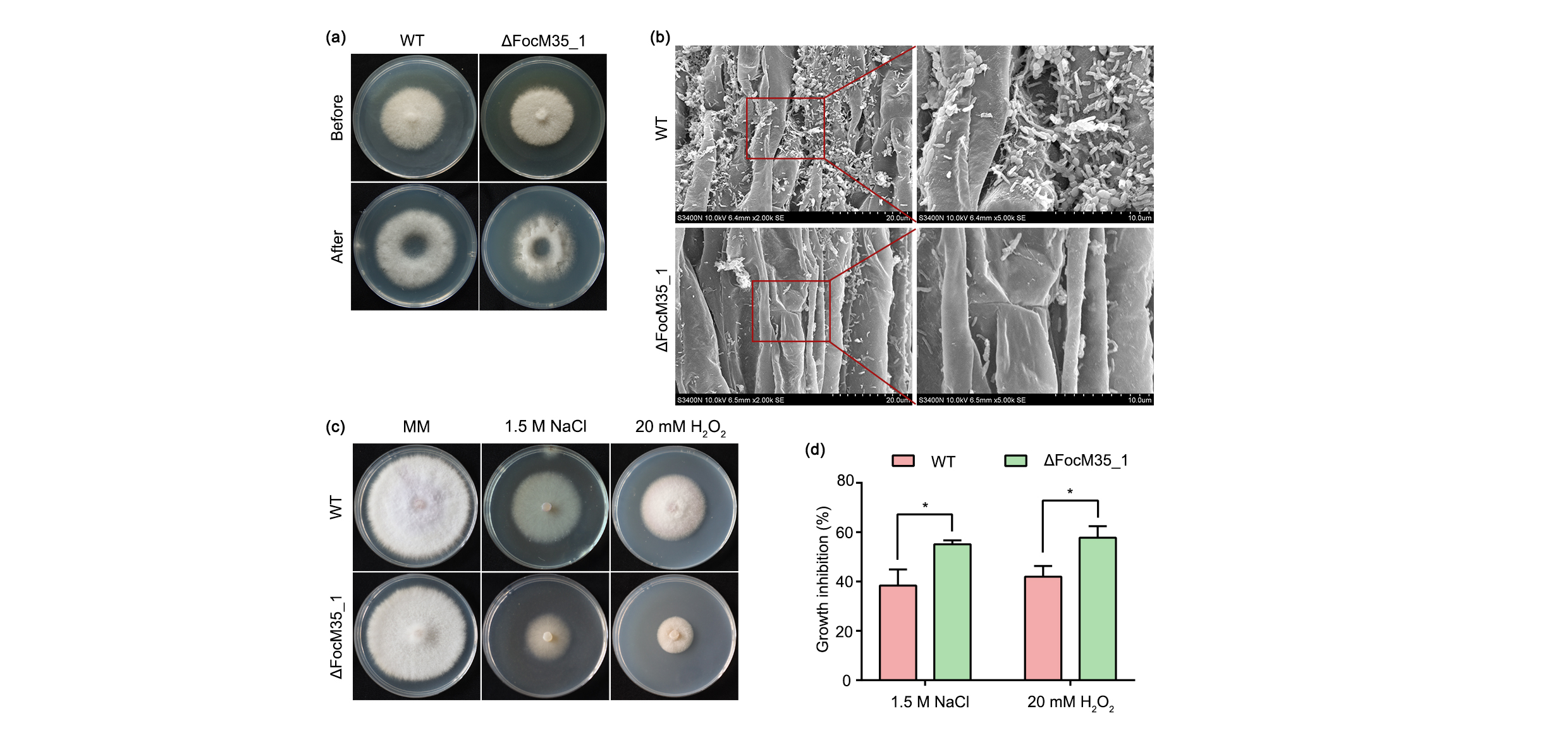

Supplement: Supplementary file 1 [file pathogens-10-00670-s001.zip › supplementary files/Figure S4. stress.jpg]

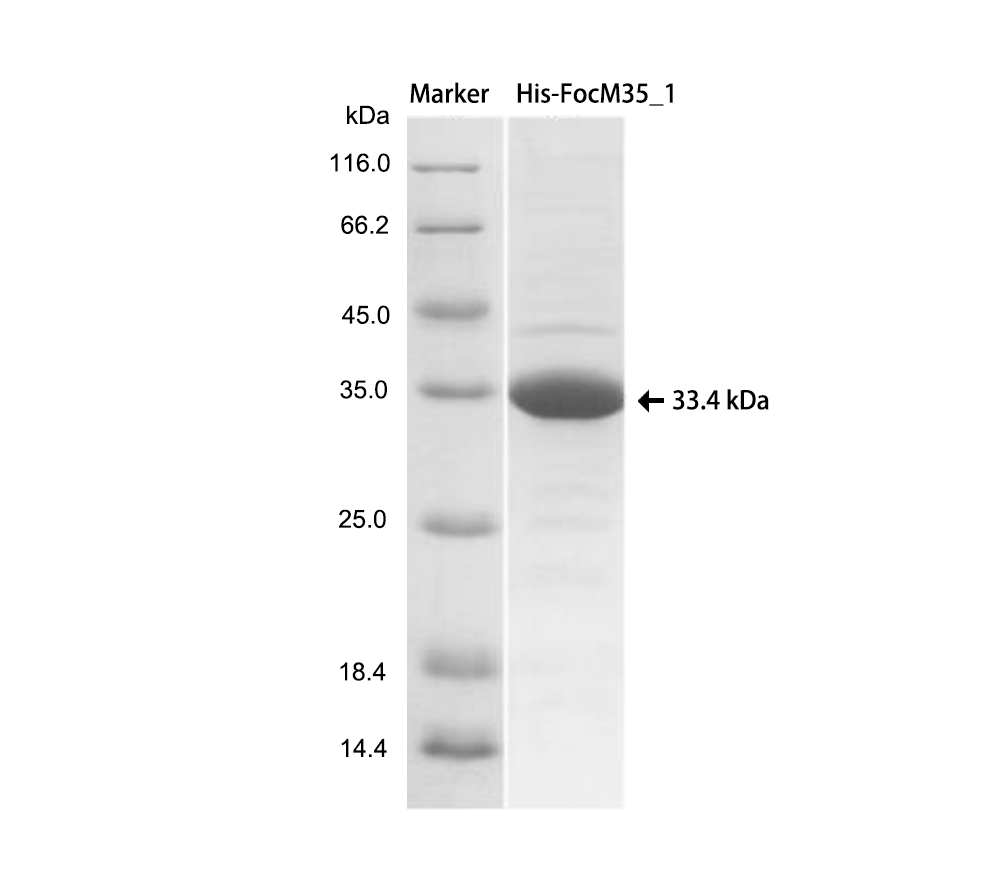

Supplement: Supplementary file 1 [file pathogens-10-00670-s001.zip › supplementary files/Figure S5. Purification of the recombinant protein.jpg]

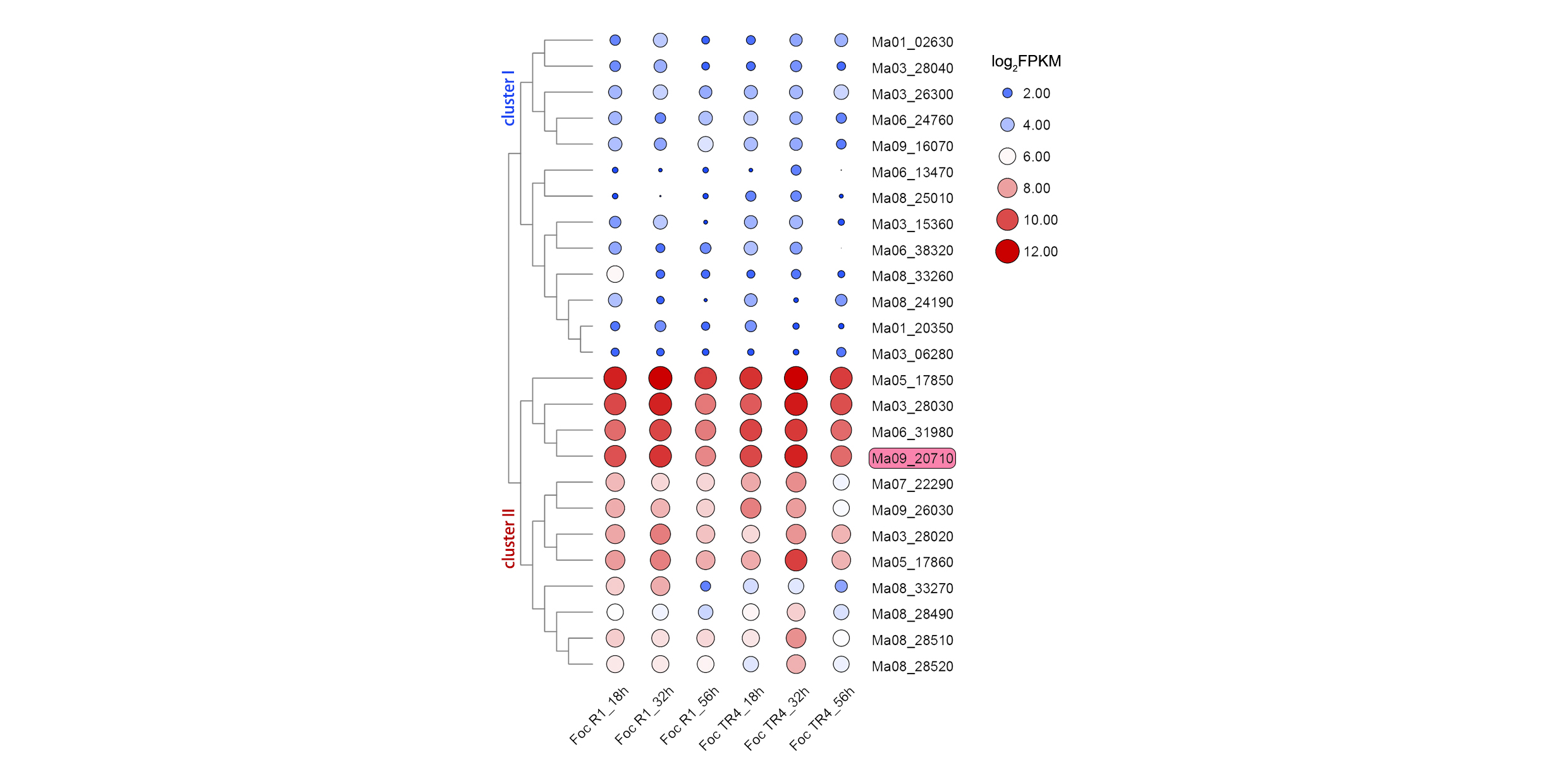

Supplement: Supplementary file 1 [file pathogens-10-00670-s001.zip › supplementary files/Figure S6. Musa Chitinase expression.jpg]
